# Characterization of Gelatin Hydrogels Cross-Linked with Microbial Transglutaminase as Engineered Skeletal Muscle Substrates

**DOI:** 10.3390/bioengineering8010006

**Published:** 2021-01-06

**Authors:** Divya Gupta, Jeffrey W. Santoso, Megan L. McCain

**Affiliations:** 1 Laboratory for Living Systems Engineering, Department of Biomedical Engineering, Viterbi School of Engineering, University of Southern California, 1042 Downey Way, DRB 140, Los Angeles, CA 90089, USA; guptad@usc.edu (D.G.); jwsantos@usc.edu (J.W.S.); 2 Department of Stem Cell Biology and Regenerative Medicine, Keck School of Medicine, University of Southern California, 1975 Zonal Ave, Los Angeles, CA 90033, USA

**Keywords:** myotube, micromolding, elastic modulus, transmittance, myogenic index, sarcomere

## Abstract

Engineered in vitro models of skeletal muscle are essential for efficiently screening drug safety and efficacy. However, conventional culture substrates poorly replicate physical features of native muscle and do not support long-term culture, which limits tissue maturity. Micromolded gelatin hydrogels cross-linked with microbial transglutaminase (gelatin-MTG hydrogels) have previously been shown to induce C21C2 myotube alignment and improve culture longevity. However, several properties of gelatin-MTG hydrogels have not been systematically characterized, such as changes in elastic modulus during incubation in culture-like conditions and their ability to support sarcomere maturation. In this study, various gelatin-MTG hydrogels were fabricated and incubated in ambient or culture-like conditions. Elastic modulus, mass, and transmittance were measured over a one- or two-week period. Compared to hydrogels in phosphate buffered saline (PBS) or ambient air, hydrogels in Dulbecco’s Modified Eagle Medium (DMEM) and 5% CO_2_ demonstrated the most stable elastic modulus. A subset of gelatin-MTG hydrogels was micromolded and seeded with C2C12 or primary chick myoblasts, which aligned and fused into multinucleated myotubes with relatively mature sarcomeres. These data are important for fabricating gelatin-MTG hydrogels with predictable and stable mechanical properties and highlight their advantages as culture substrates for engineering relatively mature and stable muscle tissues.

## 1. Introduction

Skeletal muscle is especially susceptible to adverse drug reactions because it occupies a large amount of body mass and is highly vascularized. For example, statins, azidothymidine, and hydroxychloroquine, which are prescribed for high cholesterol, HIV infection, and malaria, respectively, have each been shown to cause skeletal myopathy [1]. To evaluate drug safety at the pre-clinical stage, both in vivo and in vitro models of skeletal muscle are essential due to their tradeoffs in throughput, cost, complexity, and physiological relevance. Similarly, developing effective therapies for genetic skeletal myopathies requires a combination of in vivo models, such as transgenic animals that provide organ-level insights into disease progression [2,3], and in vitro models, which are advantageous for efficiently probing disease mechanisms on the cellular and molecular level. In vitro models of skeletal muscle tissue generated from patient-derived myoblasts are especially powerful for correlating patient-specific genotypes and phenotypes and identifying personalized drug responses for inherited skeletal myopathies [4,5].

To generate in vitro models of skeletal muscle, myoblasts have conventionally been cultured on glass or polystyrene surfaces coated with extracellular matrix (ECM) [6]. Myoblasts are then differentiated to fuse into multi-nucleated myotubes, which can be assessed using standard molecular characterization techniques or calcium imaging [7,8]. However, these conventional surfaces are incompatible with assays to quantify absolute force generation, which is important for assessing muscle function in response to genetic mutations, drugs, environmental toxins, and other perturbations. Furthermore, myoblasts fuse into myotubes with random orientation, which does not match the aligned architecture of native muscle fibers. Various methods to align myotubes in vitro have been developed [9], including culturing cells on coverslips coated with polydimethylsiloxane (PDMS) and microcontact printed with ECM proteins [10,11]. PDMS is also compatible with the muscular thin film assay for directly measuring basal, twitch, and tetanus stresses [4,12]. Another advantage of PDMS is that the rigidity can be tuned within the range of native skeletal muscle tissue (10–50 kPa) [13,14], which can improve the maturity of myotubes in vitro [13,15]. However, myotubes cultured on PDMS tend to delaminate within two weeks [16], independent of substrate rigidity [17], likely because PDMS is a highly synthetic substrate that cannot support myotube adhesion long-term. Synthetic hydrogels, such as polyacrylamide, have also been functionalized with ECM for culturing myotubes [13,18,19]. However, similar to PDMS, these substrates have a limited number of cell adhesion molecules that are important for supporting long-term culture, disease modeling, and drug testing.

Gelatin is a low-cost derivative of collagen that can be cross-linked into tunable hydrogels with elastic moduli similar to native muscle [17,20]. One approach for generating gelatin hydrogels is to modify the gelatin with methacrylate groups and activate cross-linking with photoinitiators and UV light [21,22]. Unmodified gelatin can also be cross-linked using microbial transglutaminase (MTG), an FDA-approved enzyme that covalently bonds the glutamine and lysine groups between gelatin polymers [23]. We and others have shown that gelatin hydrogels cross-linked with MTG (referred to as gelatin-MTG hydrogels) improve the long-term culture [17,24] and maturation [25] of myotubes. Gelatin-MTG hydrogels can also be micromolded to induce myotube alignment and are compatible with the muscular thin film assay to measure contractile stresses [20,26]. However, several properties of gelatin-MTG hydrogels relevant for engineering skeletal muscle tissues as reproducible in vitro models have not been characterized, including the long-term stability of their mechanical properties and optical transparency in culture-like conditions. To address this, we fabricated a variety of gelatin-MTG hydrogels, incubated them in ambient and culture-like conditions, and quantified changes in elastic modulus, mass, and transmittance over a one- or two-week period. We found that elastic modulus values of gelatin-MTG hydrogels over time was highly sensitive to culture conditions, as hydrogels incubated in DMEM in a 5% CO_2_ incubator demonstrated the most stability compared to those incubated in PBS or ambient air. Based on their stability, mechanical similarity to native muscle, and optical transparency, we selected a subset of gelatin-MTG hydrogels to micromold and seed with either C2C12 myoblasts or primary chick myoblasts and compared myotube development and sarcomere formation. Both cell types aligned with micromolded features and fused into multinucleated myotubes with well-defined sarcomeres on all gelatin-MTG hydrogels. Together, these data indicate that select formulations of micromolded gelatin-MTG hydrogels have stable elastic moduli over two weeks in culture-like conditions and promote the formation of aligned myotubes with mature sarcomeres from both immortalized and primary myoblasts, highlighting their advantages as robust culture substrates for engineered muscle tissues.

## 2. Materials and Methods

### 2.1. Measuring Gelatin-MTG Hydrogel Elastic Modulus and Mass

Solutions of 10%, 16%, 20%, and 24% *w/v* 175 g Bloom Type A porcine gelatin (Sigma, G2625, St. Louis, MI, USA) and 4%, 8%, and 12% *w/v* Activa TI transglutaminase (Ajinomoto, 1002, Chicago, IL, USA) were prepared in ultrapure water and incubated in water baths at 65 °C and 37 °C, respectively, for 30 min. Equal volumes of gelatin and MTG were combined to generate a total of twelve gelatin-MTG solutions, ranging from 5% gelatin and 2% MTG (referred to as 5–2) to 12% gelatin and 6% MTG (referred to as 12–6). The mixtures were homogenized for 30 s and degassed for 20 s in a centrifugal mixer (Thinky, Laguna Hills, CA, USA, AR-100, Laguna Hills, CA, USA), poured into 35 mm × 10 mm Petri dishes, and incubated overnight at room temperature to form a hydrogel. Cylindrical samples from each gelatin-MTG hydrogel were removed using an 8 mm biopsy punch and placed in 12-well plates. Hydrogels were then immersed in low glucose DMEM, PBS, or PBS without calcium and magnesium and placed in either a 37 °C incubator with ambient air or a 37 °C incubator with 5% CO_2_. DMEM or PBS was changed every other day.

Prior to incubation, and after 1, 4, 7, and 14 days of incubation (referred to as Day 0, 1, 4, 7, and 14, respectively), the compressive elastic modulus of hydrogel cylinders was measured using an Instron 5942 Mechanical Testing System applying 30% strain. Bluehill Universal software was used to calculate elastic modulus based on the slope of the elastic region (0–30% strain) of the stress-strain curves and the dimensions of the cylinders. The mass of the hydrogel cylinders was also measured at every timepoint by first rolling the hydrogels on a KimWipe to remove any excess liquid. For each hydrogel formulation, 3–4 independent replicates were fabricated and tested. For certain formulations, the hydrogels degraded prior to certain timepoints, preventing measurements. Any conditions with a final sample size less than three were excluded.

### 2.2. Measuring Gelatin-MTG Hydrogel Transparency

A total of 200 µL of each gelatin-MTG solution was pipetted into each well of a 96-well plate. After overnight incubation at room temperature, the plate was inserted into a Varioskan LUX Microplate Reader and percent transmittance at 640 nm was recorded, which is a common method for assessing hydrogel transparency [27]. 100 µL of PBS or DMEM was then added to each well and the plate was incubated for seven days at 37 °C in ambient air for two trials or 5% CO_2_ for two trials. Transmittance at 640 nm was then recorded again. Because the values were similar, the data for ambient air and the 5% CO_2_ incubator were pooled. For each trial, eight wells of each gelatin-MTG formulation were measured and averaged.

### 2.3. Fabrication of Micromolded Gelatin-MTG Hydrogel Coverslips

Polystyrene Petri dishes (150 mm × 15 mm) were covered with tape (Patco, 3900R, Franklin, MA, USA), placed in a 30 W Epilog Mini 24 Laser Engraver (Epilog Laser, Golden, CO, USA), and engraved into 260 mm^2^ hexagons (100% speed, 25% power, 2500 Hz) inscribed with circles (18% speed, 6% power, 2500 Hz). The circles of tape were peeled off, leaving the edges of the hexagons taped. Coverslips were plasma-treated (Harrick Plasma, PDC-001-HP, Ithaca, NY, USA) for ten minutes to increase hydrogel adhesion. Four gelatin-MTG solutions (8–2, 8–4, 10–2, 10–4) were pipetted onto the coverslips within five minutes of plasma treatment. PDMS stamps with 10 µm width, 10 µm spacing, and 2 µm depth were fabricated with standard photolithography and soft lithography techniques [28] and sonicated in 95% ethanol. PDMS stamps were then air-dried, inverted onto the hydrogel solution, and slowly pushed down at the center to ensure homogenous distribution of the hydrogel on the coverslip, while minimizing bubble formation, as previously described [17,20]. The coverslips were then incubated overnight at room temperature. The taped edges maintained an approximate height of 104 µm of the hydrogels [17,20]. Coverslips were then rehydrated with ultrapure water and stamps were slowly detached from the coverslips in the direction of the features to prevent pattern disruption. The tape on the edges was removed and coverslips were rinsed with PBS and stored in 12-well plates at 4 °C for up to a week prior to cell seeding.

### 2.4. Myoblast and Myotube Culture on Micromolded Hydrogel Coverslips

C2C12 myoblasts (ATCC) were thawed and cultured in T175 flasks in growth media (Table 1). Cells were passaged using trypsin-EDTA solution and seeded onto micromolded gelatin-MTG hydrogel coverslips at 500,000 cells per coverslip in a 12-well plate. For all experiments, only myoblasts from passages 2 to 5 were used. After 3–4 days, cells reached confluence and growth media was exchanged with differentiation media (Table 1). All media was exchanged every other day.

Day 10 chick embryos (AA Lab Eggs) were harvested by isolating and mincing the thigh muscle tissue using forceps and scalpel. Further dissociation procedures were adapted from previously reported protocols [29,30]. Briefly, four digestions in 1 mg/mL collagenase (Worthington LS004177, Lot 43K144303B) in Hank’s Balanced Salt were performed for three minutes each at 37 °C. In between each digestion, tissue was mechanically dissociated by pipetting up and down with a 10 mL serological pipette. Two consecutive 30-min pre-plating steps in T75 and T175 flasks were used to purify myoblasts from the dissected tissue. Myoblasts were then seeded onto micromolded gelatin-MTG hydrogel coverslips at 500,000 cells per coverslip in a 12-well plate in growth media (Table 1). After 3–4 days, cells reached confluence and growth media was exchanged with differentiation media (Table 1). All media was exchanged every other day.

### 2.5. Tissue Fixation and Immunostaining

After eleven days of differentiation, coverslips were rinsed three times with PBS, fixed with ice-cold methanol for ten minutes, and incubated with mouse α-actinin primary antibody (Sigma, A7811) at a 1:200 dilution for one hour. For secondary staining, tissues were incubated with goat anti-mouse antibody conjugated to Alexa Fluor 488 and 4′,6-diamidino-2-phenylindole (DAPI) at 1:200 dilutions for one hour. Coverslips were then placed on a glass slide, covered with ProLong gold antifade mountant (ThermoFisher Scientific, P36930, Waltham, MA, USA) and a glass coverslip to facilitate high resolution imaging, sealed with nail polish, and stored at −20 °C.

### 2.6. Image Acquisition and Analysis

Coverslips were imaged using a Nikon Eclipse Ti microscope with Confocal Module Nikon C2 at 20× air and 60× oil objectives. Nuclei number and myogenic index were quantified by analyzing five stitched images of six fields of view (20× objective) per tissue with a custom CellProfiler (Broad Institute, Cambridge, MA, USA) code. To calculate nuclei number, the number of nuclei per image was counted based on intensity and size thresholding of the DAPI signal and divided by the area of the stitched image. To calculate myogenic index, the number of nuclei co-localized with α-actinin was determined and divided by the total number of nuclei.

To quantify sarcomere index [31], ImageJ was used to perform two-dimensional (2D) fast Fourier transforms on twenty-five samples of 50 µm-wide areas (60× objective) within myotubes for each tissue. The data were collapsed radially to generate one-dimensional (1D) power spectra profiles, which were then normalized to have an integrated area of one. MATLAB curve fitting software was used to split the radial profiles into aperiodic (decaying exponential) and periodic (sum of multiple Gaussian functions) components. The fitted aperiodic component was subtracted from the total and the area under the periodic component was taken as the sarcomere index. Sarcomere length was calculated by drawing a line segment across six z-discs in five myotubes per tissue in ImageJ and dividing the value by five.

### 2.7. Statistical Analysis

To compare the effects of gelatin and MTG concentration on elastic modulus and transmittance for a given incubation condition, data were analyzed using two-way Analysis of Variance (ANOVA) followed by Tukey’s multiple comparisons test. For simplicity, statistical differences are only shown between formulations with the same gelatin or the same MTG concentration. To compare the effects of incubation condition on elastic modulus for a given gelatin-MTG formulation, data were analyzed using student’s *t*-test. To compare the effects of gelatin-MTG formulation on myotube morphology for a given cell type, data were analyzed with one-way ANOVA followed by Tukey’s multiple comparisons test. To compare the effects of cell type on myotube morphology for a given gelatin-MTG formulation, data were analyzed using student’s *t*-test.

## 3. Results

### 3.1. Characterization of Hydrogel Elastic Modulus and Mass over Time

**Figure 1:**
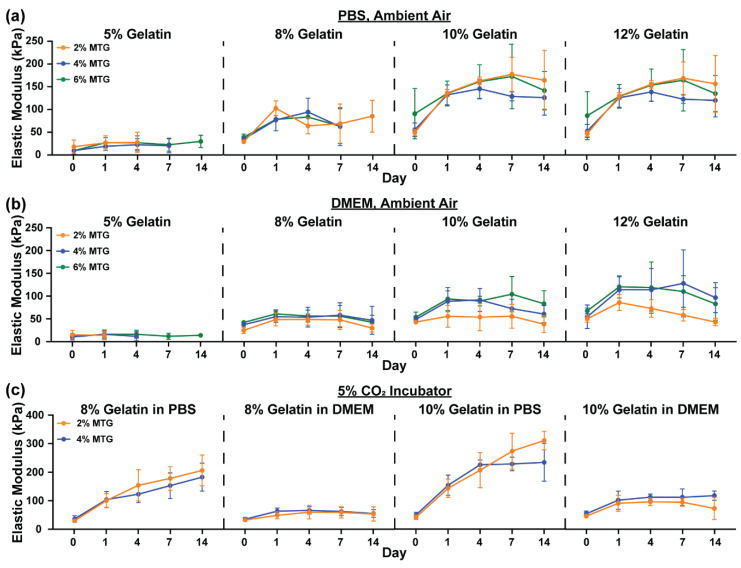
Elastic modulus values of gelatin-MTG hydrogels over 14 days in (**a**) PBS and ambient air, (**b**) DMEM and ambient air, and (**c**) PBS or DMEM and 5% CO_2_. Bars indicate standard deviation, *n* = 3–4. For some conditions, datapoints are excluded because the samples degraded prior to the measurement.

To evaluate the mechanical properties of gelatin-MTG hydrogels over time, cylinders of hydrogels were fabricated and incubated in either PBS or DMEM in a 37 °C incubator under ambient air for two weeks. The compressive elastic modulus of each hydrogel cylinder was then measured at Days 0, 1, 4, 7 and 14. Generally, the elastic modulus for hydrogels incubated in PBS increased from Day 0 to 1, followed by a plateau (Figure 1a). Within a gelatin concentration, the elastic modulus was mostly unaffected by MTG concentration. In contrast, elastic modulus consistently increased as gelatin concentration increased. In comparison to hydrogels incubated in PBS, hydrogels incubated in DMEM were generally more stable in terms of elastic modulus over two weeks (Figure 1b). In DMEM, elastic modulus was also more sensitive to MTG concentration for 10% and 12% gelatin hydrogels, as hydrogels cross-linked with 4% or 6% MTG were consistently more rigid than those cross-linked with 2% MTG. In both PBS and DMEM, 5–2 and 5–4 hydrogels degraded prior to Day 14, indicating that these are poorly suited for long-term experiments.

As micromolded gelatin hydrogels are often implemented as cell culture substrates, we next tested if incubating hydrogels in PBS or DMEM in a 5% CO_2_ incubator altered the mechanical properties of the hydrogels compared to ambient conditions. For these experiments, only 8% and 10% gelatin hydrogels cross-linked with 2% or 4% MTG were tested. Generally, the elastic modulus of hydrogels incubated in PBS continuously increased from Day 1 to 14 without reaching a plateau (Figure 1c). In DMEM, the elastic modulus was mostly stable from Day 1 to 14, indicating stark differences in elastic modulus values over time due to culture conditions.

**Figure 2:**
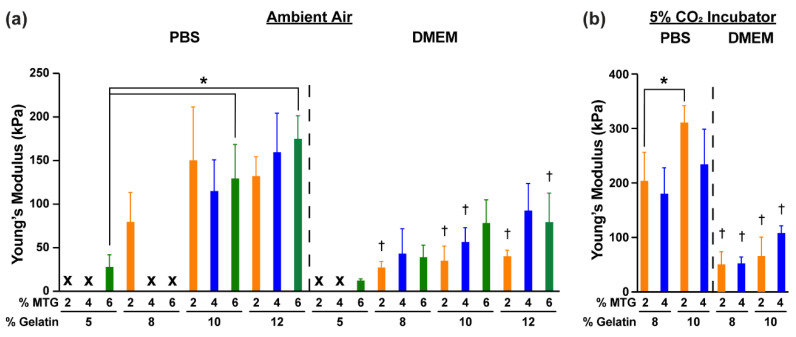
Elastic modulus values of gelatin-MTG hydrogels on Day 14 in (**a**) ambient air and (**b**) 5% CO_2_. Bars indicate standard deviation, *n* = 3–4. * indicates *p* < 0.05 for each incubation condition, based on two-way ANOVA and Tukey’s multiple comparison test. † indicates *p* < 0.05 compared to the same gelatin-MTG formulation in the same incubation environment (ambient air or 5% CO_2_) but incubated in PBS, based on student’s *t*-test. For some conditions, datapoints are excluded (indicated by X) because the samples degraded prior to the measurement.

We next compared the elastic modulus values for gelatin-MTG hydrogels incubated under different conditions at Day 14. In ambient air (Figure 2a), the elastic modulus for hydrogels incubated in either PBS or DMEM increased with gelatin concentration but was mostly unaffected by MTG concentration. For many formulations, the elastic modulus values for hydrogels incubated in DMEM were lower than those incubated in PBS. In 5% CO_2_, the elastic modulus values for all hydrogels in DMEM were significantly lower than hydrogels in PBS (Figure 2b). Together, these data indicate that the elastic modulus of gelatin hydrogels is highly sensitive to both the composition of the solution and the air, with hydrogels in DMEM and 5% CO_2_ demonstrating the highest level of stability.

**Figure 3:**
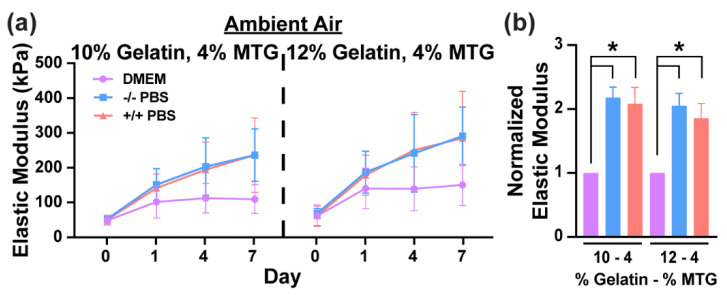
(**a**) Elastic modulus values of gelatin-MTG hydrogels over 7 days in DMEM, −/− PBS, and +/+ PBS in ambient air. (**b**) Elastic modulus values of gelatin-MTG hydrogels on Day 7 for the conditions indicated in (**a**). For each experimental replicate, the values are normalized to the DMEM condition. Bars indicate standard deviation, *n* = 3. * indicates *p* < 0.05 for each incubation condition, based on one-way ANOVA and Tukey’s multiple comparison test.

To test if calcium and magnesium salts in the PBS were responsible for differences between PBS and DMEM, 10–4 and 12–4 hydrogels were incubated in DMEM (0.2 g/L CaCl_2_, 0.98 g/L MgSO_4_), PBS with calcium (0.1 g/L CaCl_2_) and magnesium (0.1 g/L MgCl_2_) (+/+ PBS), or PBS without calcium and magnesium (−/− PBS) in ambient air. As shown in Figure 3, the elastic modulus of hydrogels incubated in +/+ PBS and −/− PBS were similar at every time point and significantly higher than hydrogels incubated in DMEM at Day 7. Thus, the differences between PBS and DMEM cannot be attributed to differences in calcium and magnesium salt content.

**Figure 4:**
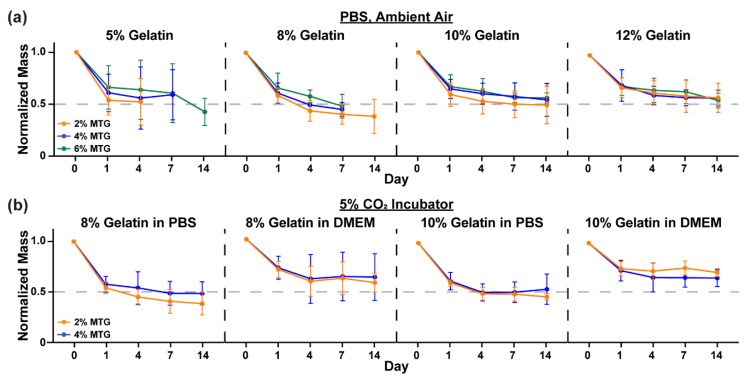
Normalized mass of gelatin-MTG hydrogels over 14 days in (**a**) PBS and ambient air and (**b**) PBS or DMEM and 5% CO_2_. Bars indicate standard deviation, *n* = 3–4. For some conditions, datapoints are excluded because the samples degraded prior to the measurement.

In addition to measuring elastic modulus at each time point, we also measured hydrogel mass. In PBS in ambient air (Figure 4a), all hydrogels lost approximately 50% of their mass from Day 0 to Day 1 before mostly plateauing, in-line with the elastic modulus data. Similar trends were observed in 5% CO_2_ in both PBS and DMEM (Figure 4b), although hydrogels in DMEM lost slightly less mass than hydrogels in PBS. Thus, the differences in the values for elastic modulus between culture conditions cannot be attributed to major differences in hydrogel mass.

### 3.2. Characterization of Hydrogel Transparency

**Figure 5:**
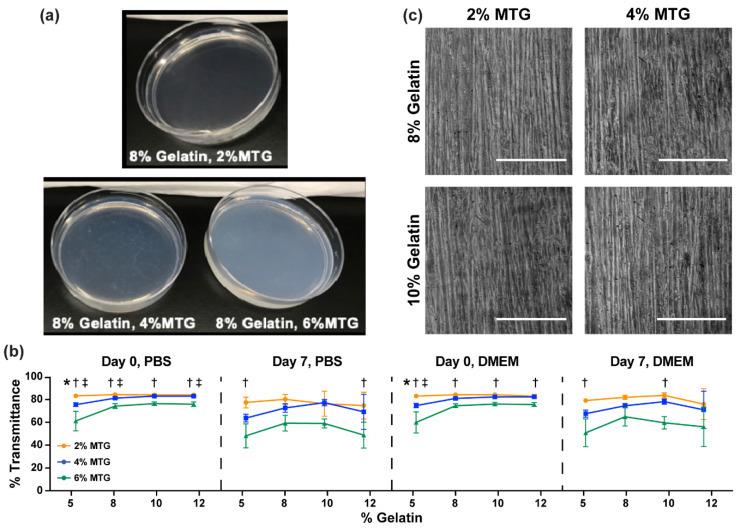
(**a**) Photographs of 8–2, 8–4, and 8–6 hydrogels on Day 0. (**b**) Percent transmittance of hydrogels in the indicated conditions. Bars indicate standard deviation, *n* = 4. * indicates *p* < 0.05 between 2% and 4% MTG, † indicates *p* < 0.05 between 2% and 6% MTG, and ‡ indicates *p* < 0.05 between 4% and 6% MTG within the same gelatin concentration, based on two-way ANOVA and Tukey’s multiple comparison test. (**c**) Images of C2C12 myotubes aligned on micromolded gelatin hydrogels with the indicated formulations. Scale bar: 200 µm.

As shown in Figure 5a, certain gelatin-MTG hydrogels are not transparent, which could obstruct routine imaging of cells. Therefore, we then evaluated hydrogel transparency by measuring the percentage of light transmitted through gelatin-MTG hydrogels incubated in either PBS or DMEM in ambient air or 5% CO_2_ (Figure 5b). As the values were similar in ambient air and 5% CO_2_, the data was combined. On Day 0, as gelatin concentration increased, transmittance generally increased from 5% to 8% and then remained relatively stable (for both PBS and DMEM: *p* < 0.05 for 5–4 compared to 10–4 and 12–4, and 5–6 compared to 8–6, 10–6, and 12–6; based on two-way ANOVA and Tukey’s multiple comparison test). However, as MTG concentration increased, transmittance decreased for most conditions, which was more apparent at Day 7 compared to Day 0. Specifically, transmittance ranged between 60–90% for 2% and 4% MTG and 40–80% for 6% MTG. There were no noticeable differences between PBS or DMEM. Thus, the transparency of gelatin-MTG hydrogels is most dependent on MTG concentration compared to gelatin concentration or incubation solution.

Next, we cultured C2C12 myoblasts on a subset of micromolded gelatin-MTG hydrogels to evaluate if the reduced transmittance disrupted imaging with a standard cell culture microscope. As shown in Figure 5c, myotubes cultured on 8% and 10% hydrogels cross-linked with 2% and 4% MTG are visible. Thus, the loss of transmittance caused by gelatin-MTG hydrogels does not significantly interfere with routine cell imaging.

### 3.3. Characterization of Myotube Maturity

**Figure 6:**
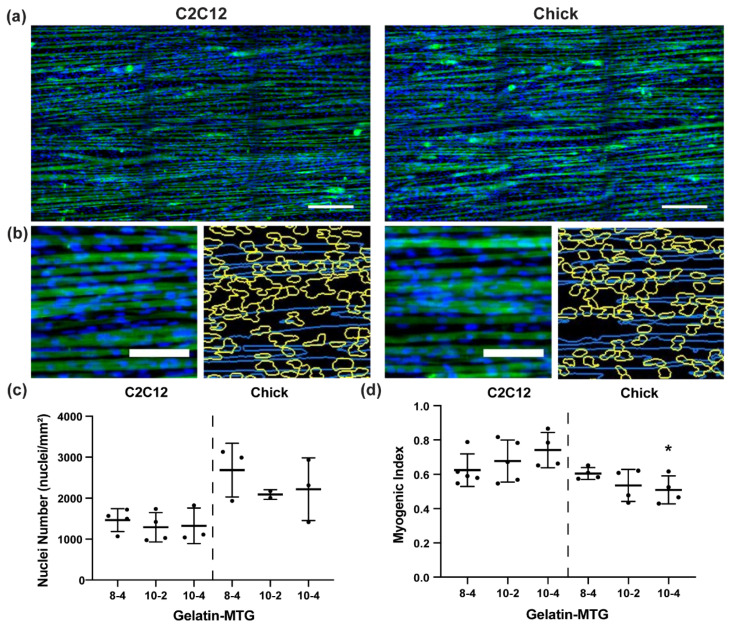
(**a**) Representative images of C2C12 and chick tissues stained for α-actinin (green) and DAPI (blue) on micromolded 10–2 gelatin hydrogels. Scale bar: 200 µm. (**b**) Stained tissues before (left) and after (right) image processing, which automatically detects nuclei (yellow outlines) and myotubes (blue). Scale bar: 100 µm. Nuclei number (**c**) and myogenic index (**d**) for C2C12 and chick tissues cultured on micromolded gelatin hydrogels with the indicated formulations. Bars indicate standard deviation, *n* = 3–4. * indicates *p* < 0.05 between C2C12 and chick tissues cultured on the same gelatin-MTG formulation based on student’s *t*-test.

To compare the ability of different gelatin-MTG hydrogel formulations to support myoblast adhesion, fusion, and differentiation into myotubes, we selected three gelatin-MTG hydrogels (8–4, 10–2, 10–4) based on their stability over two weeks, the relative similarity of their elastic modulus values to native skeletal muscle (10–50 kPa) [13,14], and their compatibility with micromolding. For example, excessive bubble formation in 8–2 hydrogels during the micromolding process, likely due to their low viscosity, limited the use of this formulation. We micromolded these hydrogels on polystyrene coverslips, seeded them with C2C12 or primary chick myoblasts, differentiated them into myotubes, and cultured for an additional 11 days. On all substrates, myoblasts fused into elongated myotubes and formed confluent muscle tissues, as shown by the representative images on 10–2 hydrogels (Figure 6a). Muscle fusion and stability was first assessed by quantifying nuclei number and myogenic index from α-actinin and DAPI stains using custom software analysis (Figure 6b). Nuclei number was slightly, but non-significantly, higher in chick tissues compared to C2C12 tissues, with no differences based on hydrogel formulation (Figure 6c). Myogenic index was similar across cell types and hydrogel formulations, except for a lower myogenic index in chick tissues compared to C2C12 tissues for 10–4 hydrogels.

**Figure 7:**
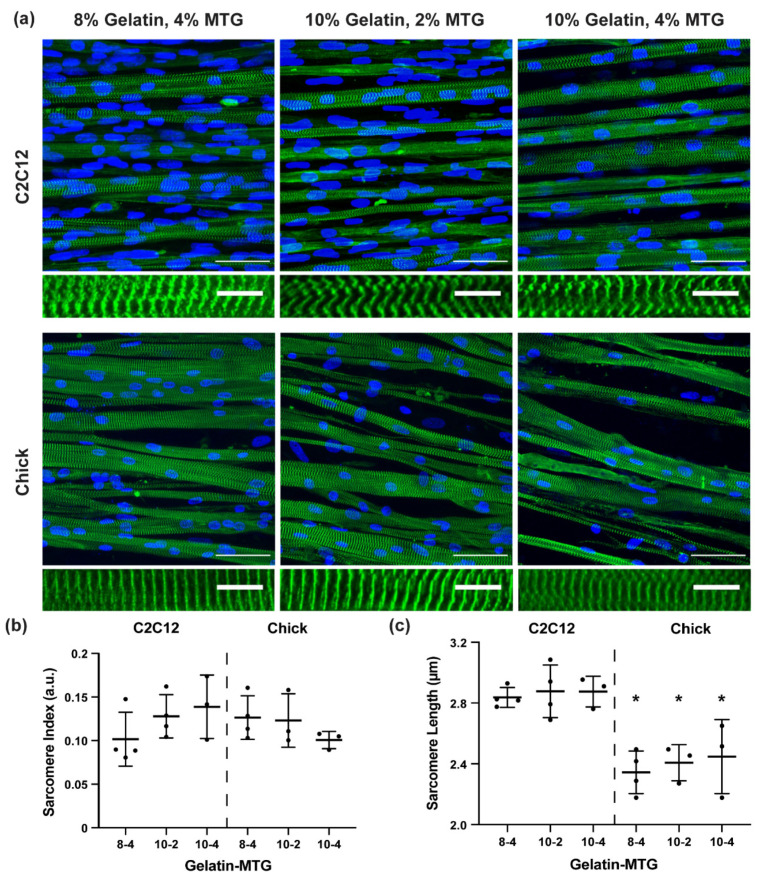
(**a**) Representative images of C2C12 and chick tissues stained for α-actinin (green) and DAPI (blue) on micromolded gelatin hydrogels with the indicated formulations. Scale bar top: 50 µm, bottom: 10 µm. Sarcomere index (**b**) and sarcomere length (**c**) for C2C12 and chick tissues cultured on micromolded gelatin hydrogels with the indicated formulations. Bars indicate standard deviation, *n* = 3–4. * indicates *p* < 0.05 between C2C12 and chick tissues cultured on the same gelatin-MTG formulation based on student’s *t*-test.

To assess sarcomere maturation, α-actinin stains and higher-resolution imaging settings were used to calculate sarcomere index and length using custom analysis software. Across all conditions, myotubes were packed with periodic and well-defined z-discs (Figure 7a). Sarcomere index was similar across all conditions (Figure 7b) whereas sarcomere length was significantly higher in C2C12 myotubes compared to chick myotubes (Figure 7c). Thus, myotube fusion and sarcomere formation was similar across all tested gelatin hydrogel formulations for both C2C12 and chick myoblasts, indicating that these substrates can support the formation of relatively mature muscle tissues from both myoblast cell lines and primary myoblasts.

## 4. Discussion

Reproducible models of engineered skeletal muscle tissues are needed to serve as testbeds for drug development and to elucidate the degenerative mechanisms behind skeletal myopathies. Towards this goal, previous studies have shown that micromolded gelatin-MTG hydrogels promote the alignment, maturity, and longevity of engineered C2C12 muscle tissues [17,25]. In this study, we systematically characterized the mechanical and optical properties of gelatin-MTG hydrogels as a function of time and incubation conditions, which is important for establishing conditions that promote hydrogel stability and reproducibility. We also demonstrated that these substrates promote robust sarcomere formation in myotubes, derived from both C2C12 and primary chick myoblasts, illustrating their broad use as substrates for engineering skeletal muscle tissues from diverse cell sources.

We found that the elastic modulus of gelatin-MTG hydrogels was highly sensitive to both the composition of the incubation solution and the concentration of CO_2_ in the air. Specifically, elastic modulus increased the most over two weeks in PBS in a 5% CO_2_ incubator, followed by PBS in ambient air. In both ambient air and 5% CO_2_, the elastic modulus values for hydrogels in DMEM were more stable compared to PBS. The most stable condition was incubation in DMEM in a 5% CO_2_ incubator, which also most closely mimics cell culture conditions. One difference between PBS and DMEM is the concentration of calcium and magnesium, which can affect enzyme activity. However, we observed no differences in the rigidity of hydrogels incubated in PBS with or without calcium and magnesium salts. This is expected because the MTG used in this study is calcium-independent [32]. Instead, the differences in stability that we observed can likely be explained by the pH sensitivity of MTG. MTG is known to function optimally around physiological pH [32,33] and gelatin-MTG hydrogels have been shown to increase in rigidity with increasing pH [34]. Physiological pH is expected to be maintained optimally in a 5% CO_2_ environment with DMEM, which includes sodium carbonate as a pH buffer. Collagen also exhibits pH-dependent increases in intermolecular forces [35], which could also contribute to hydrogel stability. Therefore, incubation in PBS and/or ambient air likely exposed hydrogels to a lower pH environment compared to incubation in DMEM and/or the 5% CO_2_ incubator, which could affect MTG activity and thus the elastic modulus of the hydrogels over time. Although cells are not cultured in PBS, these responses are important for understanding and predicting hydrogel behavior in a variety of solutions, especially since hydrogels are often fabricated or stored in PBS [23,36]. Furthermore, gelatin hydrogels have many applications beyond cell culture scaffolds, such as serving as support structures for bioprinted tissues and microfluidic devices or encapsulating drugs for controlled drug release [37], for which they may be incubated in PBS or other buffer solutions.

Culturing myotubes on substrates with defined and stable mechanical properties is important because matrix rigidity is known to regulate many myoblast phenotypes, including proliferation [38] and differentiation [13,15]. For most gelatin-MTG hydrogels incubated in DMEM in 5% CO_2_, elastic modulus increased and mass decreased from Day 0 to Day 1 and then mostly plateaued, indicating that substrates should be incubated for at least one day in culture-like conditions prior to cell seeding to ensure that the elastic modulus of the substrate is stabilized prior to muscle cell culture.

From the formulations tested in this study, 8% and 10% gelatin hydrogels are likely most suitable for culturing skeletal muscle tissues for the following reasons. First, the elastic modulus values of 8% and 10% gelatin hydrogels cross-linked with 2% or 4% MTG (approximately 50–100 kPa) are relatively similar or slightly higher than native skeletal muscle (10–50 kPa) [13,14]. Second, these hydrogels were relatively stable over two weeks in DMEM and the 5% CO_2_ incubator, similar to standard cell culture conditions. Third, 8% and 10% gelatin hydrogels cross-linked with 2% or 4% MTG had relatively high transmittance of light and thus were sufficiently transparent for routine monitoring of cells in culture. Hydrogels with 5% gelatin were generally too unstable and weak to be micromolded and handled as culture substrates. The 5% hydrogels also tended to have lower transmittance compared to hydrogels with higher gelatin concentration, which could possibly be improved by adding acetic acid to improve gelatin solubility [39]. The elastic modulus of 12% gelatin hydrogels peaked around 200 kPa under both PBS and DMEM incubation, which is an order of magnitude higher than native skeletal muscle [13]. Another limitation of 5% and 12% gelatin hydrogels is their potentially limited compatibility with contractility assays, such as traction force microscopy [19] or the muscular thin film assay [20,26]. The 5% hydrogels may not be rigid enough to sustain the basal stress of the tissue without collapsing, whereas tissues may not be strong enough to deflect 12% hydrogels.

On all substrates, C2C12 and primary chick myoblasts fused into aligned, multinucleated myotubes with robust sarcomere formation, consistent with a contractile phenotype. Overall, nuclei number, myogenic index, and sarcomere index were similar across substrates and cell types, indicating that all substrates had a similar impact on cell adhesion, viability, and fusion. Sarcomere length was significantly higher in C2C12 myotubes compared to primary chick myotubes, which is consistent with values previous reported for C2C12 myotubes [25], ex vivo chick muscle [40,41], and in vitro chick myotubes [42,43]. Therefore, gelatin-MTG hydrogels within these ranges are highly suitable for engineering relatively mature skeletal muscle tissues from multiple types of myoblasts.

Collectively, these data are an important characterization of micromolded gelatin hydrogels as substrates for engineering aligned and relatively mature skeletal muscle tissues. An important future direction is to use human myoblasts, such as those purified from muscle biopsies [4,44] or differentiated from induced pluripotent stem cells [45]. Our ability to successfully culture both C2C12 and primary chick myoblasts and differentiate them into highly striated myotubes increases the likelihood for success with other source of myoblasts. Culturing patient-derived myoblasts on micromolded gelatin hydrogels to enhance their alignment and maturation would be an especially beneficial approach for in vitro disease modeling, especially when combined with functional assays [7,8,20,26] and CRISPR/Cas9 technology for generating specific mutations or isogenic cell lines [5]. Another promising future direction is to co-culture engineered muscle tissues on gelatin hydrogels with motor neurons to model neuromuscular diseases [46,47,48], especially because elastic modulus has been shown to influence acetylcholine clustering [49]. Overall, the systematic characterization we report here is important for enabling the integration of micromolded gelatin-MTG hydrogels into many different skeletal muscle-on-a-chip platforms as new models for muscle tissue development, function, disease, injury, and drug responses in a controlled setting.

## 5. Conclusions

We report that elastic modulus values for 8–4, 10–2, and 10–4 gelatin-MTG hydrogels are similar to native skeletal muscle and relatively stable over a two-week period when incubated in culture-like conditions. The optical transparency of these hydrogels is also adequate for routine microscopy. We also show that these hydrogel formulations can be micromolded and seeded with C2C12 and primary chick myoblasts, which subsequently fuse into aligned myotubes with relatively mature sarcomere structures. Thus, 8–4, 10–2, and 10–4 gelatin-MTG micromolded hydrogels are advantageous substrates for engineering relatively stable and mature skeletal muscle tissues in vitro, which have many applications in drug screening and disease modeling.

## Figures and Tables

**Table 1:** Media components for C2C12 and chick myoblasts.

Cell Type			
C2C12 (ATCC, CRL-1772)	Growth Media		
	Component	Concentration	Product Information
	high glucose DMEM	4.5 g/L	Invitrogen, 11995-040
	fetal bovine serum	10%	Hyclone, SH3007103
	penicillin-streptomycin	1%	Lonza, 17-602E
	Differentiation Media		
	Component	Concentration	Product Information
	high glucose DMEM	4.5 g/L	Invitrogen, 11995-040
	horse serum	2%	Hyclone, SH3007103
	penicillin-streptomycin	1%	Lonza, 17-602E
	cytarabine	10 μM	Sigma, C1768
ChSKM (AA Lab Eggs)	Growth Media		
	Component	Concentration	Product Information
	low glucose DMEM	1.0 g/L	Invitrogen, 11885-084
	horse serum	10%	Hyclone, SH3007103
	chicken serum	5%	Gibco, 16110082
	vitamin B12	4 μg/mL	Sigma, V-2876
	penicillin	500 units/mL	Sigma, P-4687
	calcium chloride	3 μM	Sigma, 449709
	Differentiation Media		
	Component	Concentration	Product Information
	DMEM/F12	50%	Gibco, 11320-033
	Neurobasal	50%	Gibco, 21103-049
	N-2 Supplement	0.5×	Gibco, 17502-048
	B-27 Supplement	0.5×	Gibco, 17504-044
	vitamin C	0.1 mM	Sigma, A92902
	Glutamax	1×	
	vitamin B12	4 μg/mL	Sigma, V-2876
	penicillin	500 units/mL	Sigma, P-4687
	cytarabine	10 μM	Sigma, C1768

## Data Availability

The data presented in this study are available on request from the corresponding author.
